# The prevalence of non-invasive ventilation and long-term oxygen treatment in Helsinki University Hospital area, Finland

**DOI:** 10.1186/s12890-022-02044-5

**Published:** 2022-06-25

**Authors:** Petra Kotanen, Pirkko Brander, Hanna-Riikka Kreivi

**Affiliations:** 1 HUH Heart and Lung Center, University of Helsinki and Helsinki University Hospital, (Haartmaninkatu 4), P.O. Box 372, 00029 Helsinki, Finland; 2 Doctoral Programme in Clinical Research, University of Helsinki, Helsinki, Finland

**Keywords:** Non-invasive ventilation, Long-term oxygen treatment, Prevalence, Mortality, Chronic respiratory failure, COPD

## Abstract

**Background:**

Chronic respiratory failure (CRF) can be treated at home with non-invasive ventilation (NIV) and/or long-term oxygen (LTOT). The prevalence of these treatments is largely unknown. We aimed to clarify the prevalence and indications of the treatments, and the three-year mortality of the treated patients in the Helsinki University Hospital (HUH) area in Finland.

**Methods:**

In this retrospective study we analyzed the prevalence of adult CRF patients treated with NIV and/or LTOT on 1.1.2018 and followed these patients until 1.1.2021. Data collected included the underlying diagnosis, patient characteristics, information on treatment initiation and from the last follow-up visit, and mortality during the three-year follow-up. Patients with home invasive mechanical ventilation or sleep apnea were excluded.

**Results:**

On 1.1.2018, we had a total of 815 patients treated with NIV and/or LTOT in the Helsinki University Hospital (HUH) area, with a population of 1.4 million. The prevalence of NIV was 35.4 per 100,000, of LTOT 24.6 per 100,000 and of the treatments combined 60.0 per 100,000. Almost half, 44.5%, were treated with NIV, 41.0% with LTOT, and 14.4% underwent both. The most common diagnostic groups were chronic obstructive pulmonary disease (COPD) (33.3%) and obesity-hypoventilation syndrome (OHS) (26.6%). The three-year mortality in all patients was 45.2%. In the COPD and OHS groups the mortality was 61.3% and 21.2%. In NIV treated patients, the treatment durations varied from COPD patients 5.3 years to restrictive chest wall disease patients 11.4 years. The age-adjusted Charlson co-morbidity index (ACCI) median for all patients was 3.0.

**Conclusions:**

NIV and LTOT are common treatments in CRF. The prevalence in HUH area was comparable to other western countries. As the ACCI index shows, the treated patients were fragile, with multiple co-morbidities, and their mortality was high. Treatment duration and survival vary greatly depending on the underlying diagnosis.

## Background

Respiratory failure can be classified to two main types, hypercapnic and hypoxemic respiratory failure. Home mechanical ventilation (HMV) is a well-established treatment for hypercapnic chronic respiratory failure (CRF). It can be delivered either as non-invasive ventilation (NIV) with a mask or mouthpiece, or invasively via a tracheostomy (home invasive mechanical ventilation = HIMV). Hypoxemic CRF patients are treated with long-term oxygen treatment (LTOT). If the patient has both hypercapnia and hypoxemia the treatments can be combined. Various underlying diseases can cause CRF. Common diseased leading to hypercapnic CRF are chronic obstructive pulmonary disease (COPD), neuromuscular diseases (NMD), restrictive chest wall diseases (RCWD), and obesity-hypoventilation syndrome (OHS). Hypoxemic CRF in turn is most commonly seen in interstitial lung diseases, diseases leading to cor pulmonale and COPD with emphysema.

NIV treatment has been shown to improve patients symptoms and health status, and also survival in OHS, amyotrophic lateral sclerosis (ALS) and RCWD [1]. In COPD, the benefits have been debatable. However, NIV has recently been shown to improve survival and reduce readmissions and exacerbations [2–4]. There are international guidelines for initiation of NIV for COPD [5, 6] and OHS [7–9], but these guidelines do not cover all the diseases for which NIV is commonly used. In real life, the patients using NIV are a heterogeneous group with different underlying causes for hypercapnic CRF, different comorbidities and highly variable life expectancies. LTOT is recommended in international and Finnish guidelines for patients with chronic, severe resting hypoxemia [10–12]. In palliative care LTOT is commonly used for hypoxemic patients whose dyspnea is relieved with oxygen treatment.

The prevalence of non-invasive ventilation has been growing strongly over the last few decades [1, 13]. Home ventilators have taken great technical leaps and their costs have decreased [14]. A growing number of studies reports benefits on telemonitoring NIV home titration and follow-up [15]. Patient groups treated with NIV have also changed. Previously, RCWD patients were the predominant group, but now COPD and OHS patients are often the largest patient groups [16]. In Finland, as in many other countries, the current prevalence of NIV and LTOT is largely unknown. In 1.1.2019 the prevalence of HIMV in Finland was 2.0 per 100,000 for the whole country. In the Helsinki University Hospital area the prevalence was 1.5 per 100,000 [17]. The HIMV patients comprise only a tiny minority of all HMV patients. The aim of this study was to clarify the prevalence of NIV and LTOT in the Helsinki University Hospital area, consisting of a population of 1.4 million [18]. We also wanted to analyze the characteristics of NIV and LTOT patients and their three-year mortality.

## Methods

### Study design and statistical analysis

This study was a register-based retrospective, cross-sectional study. We included adult patients undergoing NIV and/or LTOT treatment on 1.1.2018 in the study. Under 16 years old patients treated with NIV and/or LTOT, and all patients with HIMV were excluded. Also, patients with only sleep apnea (i.e., without concomitant CRF), treated with NIV or adaptive servo ventilator (ASV), were excluded. On January 1^st^ 2018 we had 22 patients treated with HIMV, roughly 170 sleep apnea only patients treated with NIV and 130 treated with ASV.

Information collected from the patient records included prevalence of NIV and/or LTOT on 1.1.2018, basic patient characteristics and clinical data, i.e. age, diagnosis, treatment modality and duration, and data on treatment initiation and on the last follow-up visit from 1.1.2018 to 1.1.2020. The three-year mortality, from 1.1.2018 to 1.1.2021, and age-adjusted Charlson co-morbidity index (ACCI) [19, 20], on 1.1.2018, were calculated.

We divided the patients into eight diagnostic groups: COPD, NMD, RCWD, OHS, interstitial lung disease (ILD), heart disease, cancer, and miscellaneous. In the NMD group, 38.1% of the patients had ALS. The miscellaneous group was very heterogenous including patients with e.g. bronchomalacia, pulmonary hypertension and bronchiectasis. Due to the small number of patients, the heart and cancer patients are included in the miscellaneous group in the tables. The patients who underwent LTOT with their NIV were included in the NIV group, if not otherwise clearly stated. Consequently, the LTOT group consists of patients who underwent LTOT only. Spirometry and BMI were maximum ± one year from the diagnosis. In the ACCI a higher score indicates a higher mortality risk.

Patient data were collected from all the seven pulmonary departments of the HUH area hospitals. On 31.12.2017 the adult population of the HUH area was 1.4 million people. According to Finnish legislation, patients’ consent is not required for register studies in Finland [21]. Thus, only the ethics committee approval was required. The Medical Ethics Committee of the Hospital District of Helsinki University approved the study protocol (study number HUS/88/2018). Statistical analysis was conducted using SPSS (IBM SPSS Statistics, version 25). Results are shown mainly as N (%) or mean values ± SD. We considered *P* values < 0.05 statistically significant. Diagnostic groups’ comparison was done by t-test, Mann–Whitney test, and chi-square test.

## Results

### Prevalence

On January 1st 2018 there were in total 815 CRF patients being treated with NIV or LTOT or both. The prevalence of NIV and/or LTOT was 60.0 per 100,000 inhabitants. For NIV treatment, the prevalence was 35.4 per 100,000. This includes patients who underwent LTOT with their NIV. The prevalence for LTOT only was 24.6 per 100,000. The prevalence was greatest in the COPD group and lowest in the RCWD group (Table 1).

**Table 1:** Prevalence of NIV and long-term oxygen treatment in Helsinki University Hospital Area on 1.1.2018

Prevalence per 100,000*	All patients	COPD	NMD	RCWD	OHS	ILD	Misc**
NIV (N = 362)	26.7	3.4	6.0	2.4	13.3	0.1	1.6
LTOT (N = 335)	24.6	12.4	0.1	0.4	0.2	5.1	6.3
NIV + LTOT (N = 118)	8.7	4.1	0.1	1.1	2.5	0.1	0.7
All NIV and/or LTOT treatments (N = 815)	60.0	20.0	6.3	4.0	16.0	5.3	8.5

COPD, chronic obstructive pulmonary disease; NMD, neuromuscular disease; RCWD, restrictive chest wall disease; OHS, obesity-hypoventilation syndrome; ILD, interstitial lung disease; Misc, miscellaneous; NIV, non-invasive ventilation; LTOT, long-term oxygen treatment

*Children under 16, patients with invasive mechanical ventilation or sleep apnea are excluded from the study

**Misc group includes heart disease and cancer patients

### Patient characteristics

**Fig. 1:**
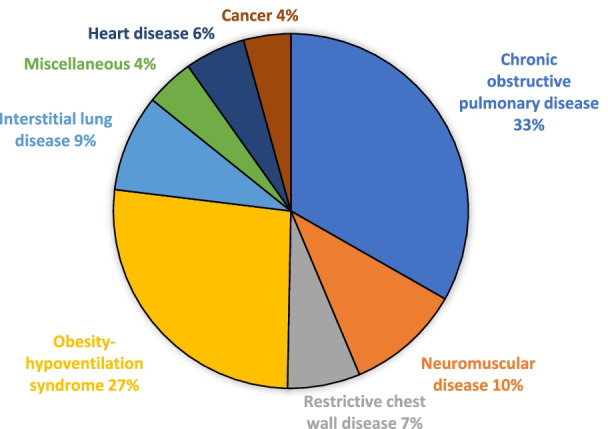
Diagnostic groups of all 815 NIV and LTOT patients on January 1st 2018. NIV, non-invasive ventilation; LTOT, long-term oxygen treatment; COPD, chronic obstructive pulmonary disease; NMD, neuromuscular disease; RCWD, restrictive chest wall disease; OHS, obesity-hypoventilation syndrome; ILD, interstitial lung disease; Misc, miscellaneous

**Fig. 2:**
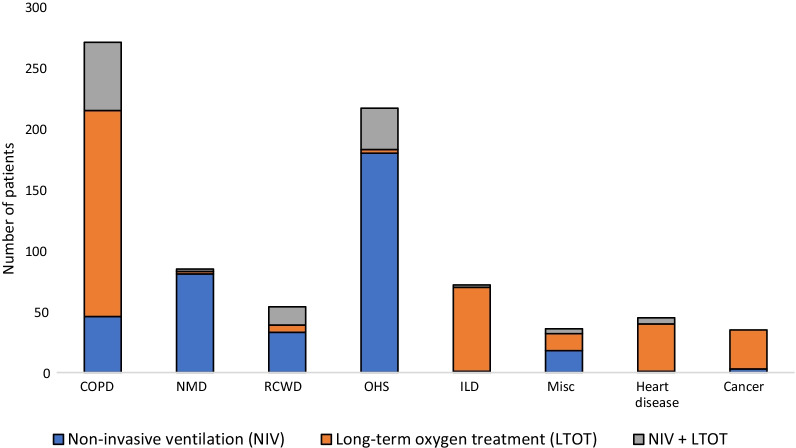
Amount of NIV and LTOT treatments in diagnostic groups on 1.1.2018. NIV, non-invasive ventilation; LTOT, long-term oxygen treatment; COPD, chronic obstructive pulmonary disease; NMD, neuromuscular disease; RCWD, restrictive chest wall disease; OHS, obesity-hypoventilation syndrome; ILD, interstitial lung disease; Misc, miscellaneous

The patient characteristics are presented in Tables 2 and 3. The largest diagnostic groups were COPD and OHS (Fig. 1). The specific characteristics of COPD patients are shown in Table 4. According to the GOLD severity classification, 38.7% of our patients had severe and 33.6% very severe COPD. The majority of OHS patients had concomitant sleep apnea (81.3%) and their average apnea-hypopnea index (AHI) was 64.6 ± 33.2. The patients were on average 68.2 ± 14.4 years old, with a slight male predominance (53.0%), and with a history of 33.2 ± 18.7 pack years. The mean predicted FEV1 was 51.4 ± 21.2% and FVC 65.6 ± 21.2%. Average BMI was 35.9 ± 13.3, ranging from 10.0 to 89.9 kg/m2. The median ACCI was 3.0. Half of the treatment initiations were performed electively, 28.3% at a pulmonary ward and 21.0% at a pulmonary outpatient clinic, and the rest during an acute exacerbation. NIV and LTOT treatment amounts in diagnostic groups are shown in Fig. 2.

**Table 2:** Patients' characteristics

	All patients	COPD	NMD	RCWD	OHS	ILD	Misc*
Patients N (%)	815 (100%)	271 (33.3%)	85 (10.4%)	54 (6.6%)	217 (26.6%)	72 (8.8%)	116 (14.2%)
Men N (%)	432 (53.0%)	153 (56.5%)	51 (60.0%)	27 (50.0%)	125 (57.6%)	38 (52.8%)	38 (32.8%)
Age at 1.1.2018 (mean ± SD), years	68.2 ± 14.4	74.5 ± 8.0	52.4 ± 17.6	67.6 ± 15.2	64.0 ± 11.6	74.8 ± 13.0	69.3 ± 16.8
FEV _1 _(% predicted)**	51.4 ± 21.2	41.4 ± 17.7	56.8 ± 27.1	41.5 ± 15.9	56.6 ± 14.9	63.5 ± 19.7	65.7 ± 23.9
FVC (% predicted)**	65.6 ± 21.2	67.6 ± 20.4	56.5 ± 26.1	44.2 ± 16.5	63.1 ± 15.1	66.8 ± 21.8	75.7 ± 23.3
Smoking %: Never-/ex-/current-smoker**	31.0/61.7/7.3	2.2/93.0/4.8	70.4/22.5/7.0	62.5/33.3/4.2	32.2/50.5/17.3	40.8/57.7/1.4	53.6/46.4/0.0
ACCI on 1.1.2018 (mean ± SD)	3.3 ± 3.1	4.6 ± 2.8	0.6 ± 1.4	1.8 ± 2.4	2.7 ± 2.6	2.9 ± 2.7	4.7 ± 3.7
BMI (mean ± SD) kg/m^2^**	35.9 ± 13.3	28.3 ± 9.2	25.3 ± 8.0	27.9 ± 7.1	47.3 ± 10.8	29.0 ± 6.5	28.7 ± 7.1
DNR decision made %**	50.2	72.3	30.6	31.5	22.1	69.4	58.6
Elective treatment initiation %***	50.7	40.1	90.3	51.1	54.5	48.6	40.7

COPD, chronic obstructive pulmonary disease; NMD, neuromuscular disease; RCWD, restrictive chest wall disease; OHS, obesity-hypoventilation syndrome; ILD, interstitial lung disease; Misc, miscellaneous; FEV_1_, forced expiratory volume; FVC, forced vital capacity; ACCI, age-adjusted Charlson co-morbity index; BMI, body mass index; DNR, do not resuscitate

*Misc group includes heart disease and cancer patients

**Information maximum ± one year from treatment initiation

***Includes initiations at the pulmonary outpatient clinic and pulmonary ward

**Table 3:** Age at treatment initiation and treatment durations of NIV and LTOT

Years (mean ± SD)	All patients	COPD	NMD	RCWD	OHS	ILD	Misc*
Age at NIV initiation	58.7 ± 15.5	68.8 ± 7.9	46.9 ± 18.3	57.9 ± 17.3	59.8 ± 11.5	62.8 ± 7.8	52.2 ± 17.7
Age at LTOT initiation	71.0 ± 12.0	72.0 ± 8.2	73.4 ± 17.5	65.3 ± 19.3	67.3 ± 9.2	73.5 ± 13.5	70.2 ± 15.9
NIV-treatment duration**	7.0 ± 5.0	5.3 ± 3.5	8.4 ± 6.9	11.4 ± 7.2	6.5 ± 3.4	6.1 ± 2.8	6.4 ± 4.5
LTOT-treatment duration**	4.9 ± 4.2	5.1 ± 3.3	5.6 ± 5.3	7.8 ± 6.4	6.0 ± 3.6	3.4 ± 3.1	4.7 ± 5.6
Lifetime of deceased patients (N = 368)	75.4 ± 11.5	77.4 ± 8.1	61.4 ± 16.8	77.4 ± 13.5	72.2 ± 8.7	77.0 ± 1.8	76.7 ± 12.6

NIV, non-invasive ventilation; LTOT, long-term oxygen treatment; COPD, chronic obstructive pulmonary disease; NMD, neuromuscular disease; RCWD, restrictive chest wall disease; OHS, obesity hypoventilation syndrome; ILD, interstitial lung disease; Misc, miscellaneous

*Misc includes heart disease and cancer patients

**Treatment duration until 1.1.2021 or death

**Table 4:** Specific data of COPD and OHS patient groups on 1.1.2018

	COPD patients with NIV* (N = 102)	COPD patients with LTOT (N = 169)	*p* value
Age at 1.1.2018 (years ± SD)	71.2 ± 7.9	75.7 ± 7.7	*p* < 0.001
ACCI (mean ± SD)	3.8 ± 2.4	5.1 ± 2.9	*p* < 0.001
Mortality (%) during 1.1.2018–1.1.2021	47.5	69.8	*p* < 0.001
Concomitant sleep apnea N (%)	32 (31.4%)	19 (11.2%)	*p* < 0.001
BMI (mean ± SD) kg/m^2^	30.5 ± 8.3	26.8 ± 9.6	*p* = 0.002
AHI (mean ± SD)**	28.7 ± 23.0	20.3 ± 17.3	*p* = 0.306

COPD, chronic obstructive pulmonary disease; NIV, non-invasive ventilation; LTOT, long-term oxygen treatment; OHS, obesity hypoventilation syndrome; ACCI, age-adjusted Charlson co-morbity index; BMI, body mass index; AHI, apnea-hypopnea index

*Includes patients who underwent LTOT with their NIV

**AHI counted for patients with concomitant sleep apnea. The information was known for 31.4% of the NIV treated and 11.2% of the LTOT treated COPD patients

### Mortality

**Fig. 3:**
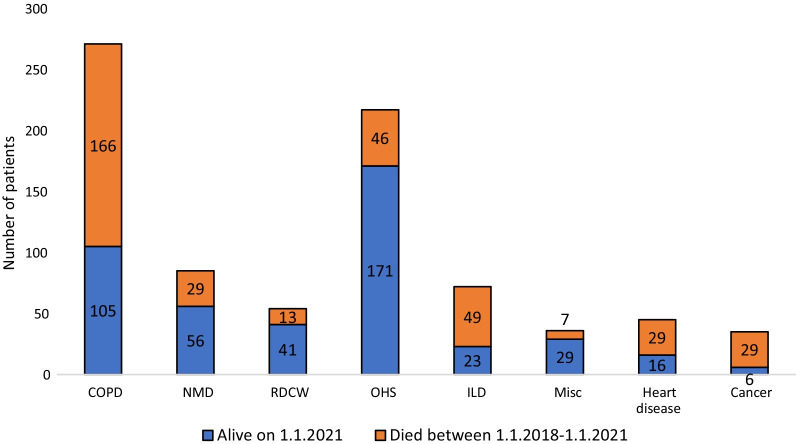
Patients’ survival during follow-up 1.1.2018–1.1.2021. COPD, chronic obstructive pulmonary disease; NMD, neuromuscular disease; RCWD, restrictive chest wall disease; OHS, obesity-hypoventilation syndrome; ILD, interstitial lung disease; Misc, miscellaneous

During the three-year follow-up (1.1.2018–1.1.2021), 45.2% of the patients died (Fig. 3). The mortality was highest in the ILD (68.1%) and COPD (61.3%) groups. In COPD patients, the mortality was greater in the non-obese patients (BMI ≤ 30 kg/m^2^) compared to the obese patients (BMI ≥ 30 kg/m^2^), 60.4% and 50.8%, but it did not reach statistical significance (*p* = 0.105). However, the BMI was missing for 45.8% of the COPD patients. In cancer and heart disease groups, the mortality was 82.9% and 64.4%. However, in these groups the treatment was largely palliative as 62.2–74.3% had a do not resuscitate (DNR) decision. If these two groups were excluded, then the overall three-year mortality was 42.0%.

Comparing treatment modalities, mortality in LTOT patients was 69.3% and in NIV patients 28.3%. If the deceased heart disease and cancer patients were excluded from the LTOT group, then the mortality in LTOT patients was 53.3%. When NIV patients were divided into NIV only and NIV with LTOT groups, their mortalities were 22.9% and 44.9%. Over half of the patients died in a hospital (61.7%), with a minority dying at home (23.0%), in a palliative care center (10.0%), or in a sheltered home (5.3%).

### Treatment durations and settings, blood gas values

The age of the patients at treatment initiation and treatment durations are shown in Table 3. On 1.1.2018 the average NIV duration for the living patients was 7.8 ± 6.2, and for the deceased 5.1 ± 3.9 years before death. For NIV treated COPD patients, the corresponding figures were for the living 5.8 ± 3.0, and for the deceased 4.4 ± 3.6 years. For LTOT patients, the durations were 7.1 ± 5.4 and 3.7 ± 2.9 years.

The baseline arterial and transcutaneous blood gas values are shown for all patients, COPD and OHS groups in Table 5. For LTOT only patients the initial partial pressure of arterial blood oxygen (PaO2) was 7.2 ± 1.1 kPa and PaCO2 was 5.3 ± 1.1 kPa. At the follow-up visit the NIV treated patients’ PaCO2 was 5.8 ± 0.9 kPa, but the information was missing from 65% of the patients.

**Table 5:** Baseline average arterial and transcutaneous blood gas values

Mean ± SD	All patients NIV initiation (N = 363)	All patients LTOT initiation (N = 335)	All patients NIV + LTOT initiation (N = 117)	COPD patients NIV initiation (N = 46)	COPD patients LTOT initiation (N = 169)	COPD patients NIV + LTOT initiation (N = 56)	OHS patients NIV initiation (N = 214)
ABG PaO2 (kPa)	8.7 ± 2.3	7.2 ± 1.1	7.4 ± 1.8	7.7 ± 1.5	7.2 ± 1.0	7.0 ± 1.3	8.2 ± 2.0
ABG PaCO2 (kPa)	7.9 ± 2.6	5.3 ± 1.1	7.9 ± 2.8	8.4 ± 2.5	5.4 ± 1.2	7.4 ± 1.8	8.0 ± 2.4
SpO2%	86.6 ± 10.2	86.2 ± 5.7	83.3 ± 13.2	86.2 ± 4.6	85.9 ± 4.4	80.8 ± 20.2	83.3 ± 11.2
PtcCO2 (kPa)	7.1 ± 1.1	5.0 ± 1.1	7.1 ± 1.2	7.2 ± 0.7	5.1 ± 0.7	7.0 ± 1.1	7.3 ± 1.0

NIV, non-invasive ventilation; LTOT, long-term oxygen treatment; COPD, chronic obstructive pulmonary disease; ABG, arterial blood gas test; PaO2, partial pressure of arterial blood oxygen; PaCO2, partial pressure of arterial blood carbon dioxide; kPa, kilopascal; SpO2, oxygen saturation; PtcCO2, transcutaneous partial pressure of carbon dioxide

The mean ± SD daily NIV use was 7.5 ± 4.3 h/night. The majority (72.9%) of the patients used NIV ≥ 90% of the nights; the median value for used nights was 99.0%. In surviving COPD patients, the mean ± SD daily NIV use was 7.8 ± 3.7 h/night and 78.0% used NIV ≥ 90% of the nights. For the deceased, the corresponding values were 5.7 ± 4.4 h/night, 64.3% used NIV ≥ 90% of the nights. However, information on NIV use was missing in 22% (hours/night) and 25% (nights used) of the patients. The mean (± SD) LTOT oxygen flow was 2.4 ± 1.3 l/min. Information on LTOT daily use was not available.

**Fig. 4:**
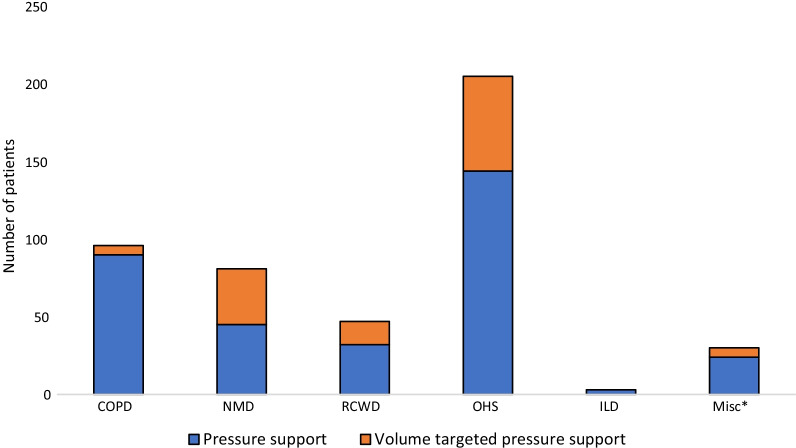
NIV-modes at treatment initiation. COPD, chronic obstructive pulmonary disease; NMD, neuromuscular disease; RCWD, restrictive chest wall disease; OHS, obesity-hypoventilation syndrome; ILD, interstitial lung disease; Misc, miscellaneous; NIV, non-invasive ventilation. *Misc group includes heart disease and cancer patients

The majority (73.4%) of patients had NIV in pressure support mode (Fig. 4). The mean (± SD) initiation pressures (cmH2O) were IPAP 13.7 ± 3.1, and EPAP 6.7 ± 2.4, and at the last control IPAP 15.2 ± 3.6 and EPAP 7.5 ± 2.6, respectively.

## Discussion

In our retrospective study, the prevalence of NIV was 37.0 per 100,000 inhabitants and of LTOT 24.6 per 100,000 in the Helsinki University Hospital area on 1.1.2018. Altogether, the prevalence was 60.0 per 100,000 and we had 815 patients in a population of 1.4 million. The three-year mortality was high at 45.2%, but depended largely on the underlying disease.

Over the last few decades, NIV prevalence has grown rapidly and the diagnostic groups treated with NIV have changed. Previously, chronic hypercapnic respiratory failure due to mainly restrictive diseases, e.g. chest wall disorders, post-polio syndrome, sequelae of tuberculosis, and neuromuscular disorders, was treated with NIV [22]. In our study, COPD and OHS were the largest diagnostic groups treated with NIV, and restrictive diseases and NMD were minorities, 6.5% and 12.3%, respectively. This is in line with the Lake Geneva area study and with the Swedish national register for patients on Long Term Oxygen Therapy and Home Mechanical Ventilation (Swedevox) [16, 23].

The prevalence for NIV and LTOT treatments in HUH are comparable to other recent studies. The NIV prevalence in Geneva, Switzerland was 37.9 per 100,000 inhabitants (2017) [16], in Norway 47 per 100,000 (2019, Norwegian National registry for long-term ventilation) and in Sweden 33 per 100,000 (2018) [23]. In Sweden the prevalence of LTOT was 22.8 per 100,000 (2018) and in Denmark 48.1 per 100,000 (2010) [23, 24].

In many countries the prevalence for LTOT is greater than the NIV prevalence. However, studies on LTOT prevalence are more scarce and many of them are older than the NIV studies. In Sweden, the Swedevox register follows the prevalences of NIV and LTOT yearly. The Swedish LTOT prevalence was at its peak at 2014 (27 per 100,000) and has slightly decreased during the recent years (22.8 per 100,000, 2018). During the same time period, the NIV prevalence has in turn risen from 29 to 33 per 100,000 [23]. We presume the trend is similar in Finland. The prevalences of both treatments vary also largely in Sweden and in different HUH hospitals, probably also nationally in Finland. The guidelines for LTOT are similar in Finland compared to international guidelines [10–12] and they are conscientious followed. In Finland, continuous smoking is a contraindication to LTOT, but NIV can be initiated despite smoking. If the patient has hypoxemia and hypercapnia, the patient is primarily given NIV alone and if the hypoxemia is not improved then LTOT is added to NIV. LTOT is not given alone to patients with hypercapnia. In most of the Finnish hospitals only pulmonologist can prescribe LTOT. These regulations and practices might explain our lower amount of LTOT treatments, especially in the COPD patient group.

The overall mortality was high in our population, 45.2% in three years. The highest mortality was in the cancer, ILD, and heart disease groups. This is expected, as the diseases generally have low prognosis. In our study these patients had high ACCI scores, and the majority had made a DNR decision. As the number of cancer and heart disease patients was small in our study, these numbers should be considered only indicative. Our NIV patients’ mortality, 28.3%, was in line with a recent, large European study, where the three-year mortality was 31.3.% [25]. Their overall survival was better (6.6 vs. our 5.1 years), but their follow-up time was longer, and the study included 10.5% sleep apnea patients.

The survival benefit of NIV in COPD patients with hypercapnic chronic respiratory failure is not yet established, as previous randomized studies have been inconsistent. However, recent studies have shown positive impact on COPD patients’ long-term survival with NIV. In studies, NIV-treated COPD patients’ one year mortality was 12–28% and in control group 33–46% [2, 26], and three and four year mortalities, 43.9–45.5%[27–29].

Treatment durations and survival of our COPD patients were in line with other studies even though our patients were older (on average 74.5 vs 62.2–70.6 years) [2, 26–29], and had many co-morbidities. Our patients’ hypercapnia was similar to many studies [2, 27, 28]; in only one study was the hypercapnia markedly lower (48.5 mmHg = 6.5 kPa) [29]. Many of these studies were done with high-intensity NIV. Compared to these studies, our NIV pressures were remarkably lower, average follow-up IPAP 14.4 ± 2.7 cmH20 and EPAP 7.1 ± 2.3 cmH20. Despite the lower pressures, the PaCO2 decreased to on average 5.8 kPa. Due to the missing information, retrospective nature of the study, and the limited number of patients, these findings should be interpreted with caution.

There were some differences between our hypercapnic COPD patients treated with NIV and hypoxemic COPD patients treated with LTOT. The NIV-treated patients’ mortality (55.4 vs 69.8%) and ACCI (3.8 ± 2.4 vs 5.1 ± 2.9) were lower than LTOT patients. The NIV patients were younger (72.3 vs 76.1 years), more obese (BMI 30.8 vs 28.1 kg/m^2^), and unsurprisingly had a greater hypercapnia (7.9 vs 5.4 kPa). Interestingly, surviving COPD patients with NIV had better adherence to the treatment than those who deceased. It can be speculated whether the higher mortality was caused by lower NIV adherence or whether these patients already had more severe morbidity during the initiation, which led to lower adherence and higher mortality.

As with NIV, the effect of LTOT on mortality is also still under debate. In the 1980s two studies showed that LTOT reduced mortality in COPD patients with severe resting hypoxemia [30, 31]. These results led to several guidelines. In present guidelines, e.g. GOLD and the ATS guideline 2020 [11, 32], supplementary oxygen is recommended for COPD patients with severe resting hypoxemia and in the ATS guideline also for severe exertional hypoxemia. The guidelines do not suggest LTOT in mild to moderate chronic resting hypoxemia.

In a large international study in 2016, LTOT treated hypoxemic COPD patients had a three-year mortality of 19% [33]. This is a clearly better survival rate than in our study. These patients were younger (68.4 vs. 76.1 years), but with a lower FEV1 (34.4 vs 43.2% of predicted). In Sweden the one-year mortality was more similar to ours, about 40% [23]. However, the median LTOT durations were shorter in the Swedish and Danish studies, 1.4 and 1.5 years compared to our 4.9 years [23, 24].

The second largest patient group in our study was OHS. Our patients were slightly more hypercapnic (PaCO2 8.0 vs. 5.7–7.9 kPa), but otherwise the patients' characteristics were similar compared to other studies [16, 34, 35]. As expected, the mortality of our OHS patients was the lowest of the diagnostic groups (21.2%) and comparable to earlier studies, in which the five-year mortality ranged from 11% to 22.7% [8, 34–37].

Since 2018 the guidelines for OHS have changed. Now continuous positive airway pressure (CPAP) is considered the first-line treatment for ambulatory patients with OHS and concomitant severe sleep apnea [7–9]. This will diminish the amount of NIV treatments in OHS patients in coming years. In our study, over 80% of the OHS patients had concomitant sleep apnea. But as patients treated with CPAP were excluded, we were not able to compare these two treatment modes.

Treatment initiation and follow-up protocols varied greatly in our area’s clinics. Half of the initiations were performed electively (49.3%). Earlier studies have shown NIV home initiations to be non-inferior to in-hospital initiations [38]. The main reason for these variances between clinics is the different resources at the wards and outpatient clinics. We did not specifically evaluate the effect of initiation unit on the treatment results or on mortality, but no large differences were seen. Lately, due to the COVID-19 pandemic and developments in remote monitoring systems, both the initiations and follow-ups have shifted more to the outpatient clinics in our hospitals. In our opinion, the most significant factors affecting the success of the NIV treatments are the clinical skills of the medical staff and efficient treatment initiation and follow-up protocol, not the type of initiation unit itself.

Our study’s strength is the population size, 815 patients in a population of 1.4 million. This is one of the largest prevalence studies recently performed. Also, the prevalence is very reliable. In Finland all NIV and LTOT treatments are coordinated from special health care, therefore, the risk of missing patients is low. All equipment is loaned to patients free of charge. Thus, patient’s financial situation rarely affects the treatment decisions. As the study included an unselected population of patients, our study provides real-life practical information to physicians treating chronic respiratory failure.

The largest limitation in our study is the observational cross-sectional study design, which prevents analyses on treatment efficacy or on the initiation or follow-up protocols. We acknowledge that the treatment initiation criteria might have differed slightly due to patient heterogeneity and clinical decisions of the treating physicians. Due to real-life study design some values were missing from patient records. Our observations might not be directly generalizable to all other countries, where the treatment financing and patient demographics differ.

## Conclusions

Chronic respiratory failure is commonly treated with home NIV and/or LTOT and its prevalence has been rising rapidly. In our large, retrospective study the prevalence in Helsinki University Hospital area of NIV was 35.4 per 100,000, of LTOT 24.6 and of the treatments combined 60.0 per 100,000. The treated patients had multiple co-morbidities and their three-year mortality was high, 45.2%. It might be speculated that in the future the number of NIV treated hypercapnic CRF patients will continue to rise due to developments in NIV treatments and prolonged survival of these patients. On the other hand, the OHS patients’ altered treatment protocol of CPAP as first-line treatment might diminish the number of their NIV treatments.

## Data Availability

The data that support the findings of this study are available on request from the corresponding author, P. Kotanen. The data are not publicly available due to the data containing information that could compromise the privacy of research participants.

## Abbreviations

ABG: Arterial blood gas test
ACCI: Age-adjusted Charlson co-morbidity index
ALS: Amyotrophic lateral sclerosis
AHI: Apnea–hypopnea index
ASV: Adaptive servo ventilator
BMI: Body mass index
CRF: Chronic respiratory failure
COPD: Chronic obstructive pulmonary disease
CPAP: Continuous positive airway pressure
DNR: Do not resuscitate
FEV_1_: Forced expiratory volume
FVC: Forced vital capacity
HUH: Helsinki University Hospital
HIMV: Home invasive mechanical ventilation
HMV: Home mechanical ventilation
ILD: Interstitial lung disease
LTOT: Long-term oxygen treatment
NIV: Non-invasive ventilation
NMD: Neuromuscular disease
PaCO2: Partial pressure of arterial blood carbon dioxide
Pa02: Partial pressure of arterial blood oxygen
PtcCO2: Transcutaneous partial pressure of carbon dioxide
OHS: Obesity-hypoventilation syndrome
RCWD: Restrictive chest wall diseases
